# Upper Vascular Thoracic Outlet Syndrome: A Case Study

**DOI:** 10.3390/biomedicines12081829

**Published:** 2024-08-12

**Authors:** Agnieszka Wierciak-Rokowska, Agnieszka Sliwka, Mikolaj Maga, Mateusz Gajda, Katarzyna Bogucka, Pawel Kaczmarczyk, Pawel Maga

**Affiliations:** 1 Independent Researcher, Reha Centrum, Physiotherapy Practice, Orthopaedic Field, Zakopianska Street 166, 30-435 Krakow, Poland; kontakt@rehacentrum.eu; 2 Institute of Physiotherapy, Faculty of Health Sciences, Jagiellonian University Medical College, 31-126 Krakow, Poland; 3 Department of Angiology, II Chair of Internal Medicine, Jagiellonian University Medical College, 30-688 Krakow, Poland; mikolaj.maga@uj.edu.pl (M.M.); mateusz14.gajda@uj.edu.pl (M.G.); k.bogucka@uj.edu.pl (K.B.); pawel.kaczmarczyk@uj.edu.pl (P.K.); pawel.maga@uj.edu.pl (P.M.)

**Keywords:** TOS, upper thoracic outlet syndrome, nonoperative management, vascular TOS, physiotherapy in TOS

## Abstract

Thoracic outlet syndrome (TOS) is recognised in approximately 8% of the population. Vascular presentation is rare and diagnosis is often elusive due to its rarity. As episodes of TOS in the upper extremities are rare, proven protocols for rehabilitation management are lacking. The purpose of our article is to present a clinical examination protocol and a treatment protocol for patients after an episode of venous thrombosis in the upper limb (VTOS). We report the case of a middle-aged woman with right venous TOS with pain in the right upper extremity, accompanied by oedema and mild violet discolouration. The results after 10 sessions of physiotherapy were as follows: a reduction in symptoms of approximately 40%, an improvement of approximately 15% in sports performance, and an improvement of approximately 25% in work. There was also an improvement in the results of TOS provocation tests, i.e., a 50–100% improvement in pulse rate and about 30% less discolouration in the extremity. Additionally, there was a significant improvement in posture between the two sides of the upper quadrant. The results after 10 physiotherapy sessions are surprising due to chronic disease after the thrombosis episode. It appears that even after a long period of time since diagnosis, improvement is possible.

## 1. Introduction

Thoracic outlet syndrome (TOS) is defined as a nonspecific set of clinical symptoms associated with nerve and/or vascular structures within the upper thoracic aperture, specifically brachial plexus compression leading to neurophysiological abnormalities (neurogenic TOS), and venous or subclavian artery compression leading to objectively visualised vascular compression (vascular TOS) [1]. The neurogenic form occurs in 90% of TOS patients and the venous form in 3%. However, there are atypical forms of this syndrome in which there are no generally accepted diagnostic criteria, and the symptoms of the nervous system overlap with those of the vascular system [2]. Various anatomical anomalies have been suggested as causes of TOS, including narrowing of the thoracic outlet through the cervical rib (cervical rib syndrome), extra fascial bands, overloading or repositioning of scalene muscle attachments, muscle hypertrophy, asymmetry of neck and shoulder alignment, trauma to the area, sports overloading, and tumours and cysts that reduce opening space [3]. A person affected by TOS may experience pain in the shoulder and neck region radiating to the arm, paresis or paralysis of the muscles innervated by the brachial plexus, and sensory disturbances. Pulse loss is observed in the abducted upper extremity and ischaemia and oedema can occur [3]. Venous TOS (VTOS) is the result of the compression of the subclavian vein, which re-enters the thorax, passing anteriorly near the junction of the clavicle and first rib, which is further reinforced by the subclavian muscle and tendon. According to the review by Povlsen et al., VTOS can manifest itself as acute thrombosis, chronic stenosis (post-thrombosis), intermittent obstruction without thrombosis, or complete chronic obstruction [4]. The venous form of the syndrome is very often diagnosed only after a thromboembolic episode of the upper extremity, despite retrospectively reported nonspecific symptoms previously [5,6]. The available reports on conservative and surgical intervention and positive and negative treatment results do not allow for a clear determination of the applied effectiveness of therapeutic management algorithms [7]. In particular, in the venous form of the syndrome, there are no reports on the effectiveness of the physiotherapy used, which is recommended as the first line of conservative treatment of neurological TOS [4,7]. Furthermore, no studies have been performed on how physiotherapeutic management after venous thrombosis can support the recommendation for anticoagulant treatment [8].

## 2. Detailed Case Description

### 2.1. Clinical History

The team of the Department of Rehabilitation in Internal Diseases and the Department of Angiology of the Jagiellonian University Medical College in Krakow is carrying out a research project (with the consent of the Bioethics Committee no. 1072.6120.123.2023) entitled “Development of a preventive protocol to detect people at risk of venous thrombosis in the course of upper thoracic vascular syndromes. Implementing physiotherapy as a form of conservative treatment and preventive measure”. As part of the pilot study that preceded the recruitment of patients for the above research project, a 50-year-old woman was included in the study, who had a history of a venous thrombosis episode in the right upper limb. 

The thrombosis episode occurred in 2017 and was the first episode. The symptoms the patient observed during the episode were a gradual increase in swelling of the upper limb and then bruising and weakness, which gradually appeared while working on the computer. Complaints, such as swelling, have persisted until now. Additionally, the patient complained of pain in the arm, shoulder, and scapula. Occasionally, there was also awakening nocturnal paraesthesia in both hands. Activities during which there continued to be a feeling of heaviness, more bruising, and a lack of pulse were activities that involve lifting the upper limb above 120 degrees. In her history, the patient denied previous trauma and falls, surgery, and taking contraceptive pills. The sports activities she participated in were gym 1× a week, swimming 2× a week, and cycling depending on the weather about 2× a week. The patient works professionally as a physical therapist. At work, she often uses support positions when she massages clients. Occasionally, there are activities that require lifting the upper limb above the head. 

### 2.2. Clinical and Imaging Examination

**Figure 1:**
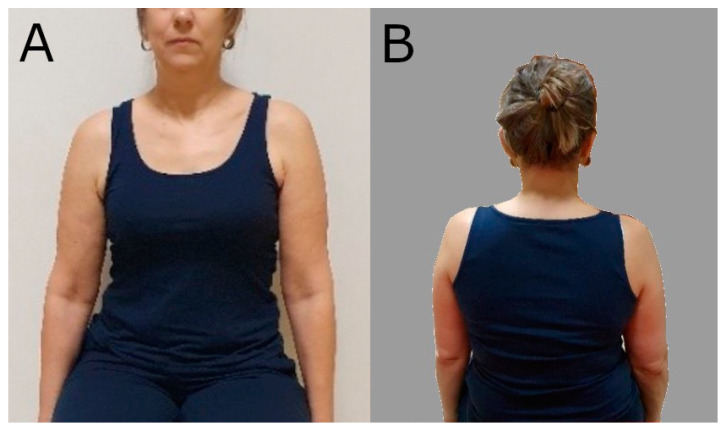
The images show lower positioning of the shoulder girdle on the right side, i.e., on the involved side. (**A**) from the front; (**B**) from behind.

The clinical/physiotherapeutic examination was divided into several stages. In the clinical examination, the alignment of the static bone points [9] and the mobility of the shoulder girdle [10], as well as neighbouring regions, i.e., cervical, thoracic, shoulder joint, scapular work, and position, were assessed (Figure 1). The variables studied were the difference in height of the acromion processes between the right and left sides of the body (measured relative to the lateral edge of the acromion process), the difference in alignment of the acromion processes in the transverse plane between the right and left sides of the body (rotation of the shoulder girdle, measured at the anterior edge of the acromion process), and the difference in alignment of the right to left shoulder–clavicular joints (measured at the level of the ACJ (acromio-clavicular joint) joint space. Subsequent measurements (4,5,6) were made of the position of the scapula, i.e., the height of the right scapula relative to the left in the frontal plane (measurement at the top of the scapula’s superior angle), the distance of the scapula’s inferior angle from the thorax (information regarding the scapula alignment in the sagittal plane), and the distance of the right scapula’s inferior angle relative to the left scapula from the spine (measurement of the degree of scapula rotation in the frontal plane, taken from the scapula’s inferior angle to the spinous process in a straight line) [9,10,11].

The next step was to assess the muscular status in these regions. This stage included neurodynamic tests, TOS provocation tests, and an assessment of the patient’s quality of life after the episode using the Quick DASH (Disabilities of the Arm, Shoulder and Hand) questionnaire [12]. As a criterion to evaluate the effectiveness of the physical therapy intervention, initial and final measurements (after 10 weeks) of static bone points as well as an evaluation of ranges of motion in individual regions such as the shoulder joint and cervical region were taken, followed by an assessment of the results from neurodynamic tests and an evaluation of soft tissue consistency in the upper body region. Changes in TOS provocation tests were also evaluated, and the results of the Quick DASH questionnaire were compared.

**Figure 2:**
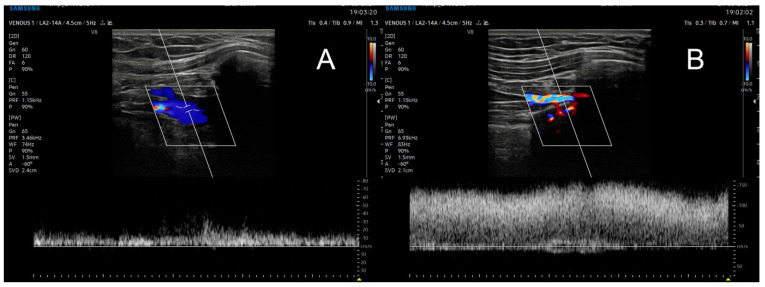
Ultrasound examination of the subclavian vein in resting position (**A**) and in abduction to 90 degrees (**B**)**,** with a high increase in flow near occlusion.

Imaging examination can take two forms. The first is the more available functional ultrasound examination, which, depending on the degree of compression by bone structures, may show narrowing and increased flow, or complete vessel occlusion depending on the degree of abduction/flexion of the upper limb in the shoulder joint (Figure 2). 

**Figure 3:**
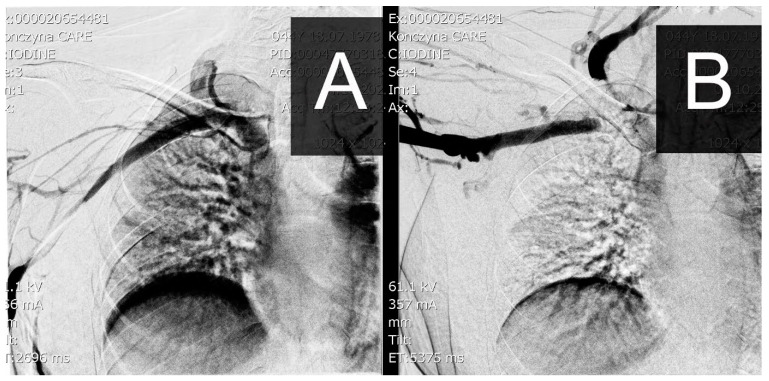
Image of a venographic examination at rest (**A**) and with the limb abducted to 90 degrees (**B**) with occlusion at the level of the clavicle/1st rib.

The second option is a functional but invasive phlebographic examination, which requires access to the peripheral veins of the examined limb and the administration of a contrast agent under diagnostic conditions. During examination, the standard anatomical positioning of the limb and abduction up to 90 degrees are used. In a venographic image, you can similarly obtain an image of stenosis or complete occlusion, as shown in Figure 3.

### 2.3. TOS Provocation Tests

TOS provocation tests were performed as part of a clinical study, using bilateral pulse rate measurements with pulse oximeters [13]. Several provocation tests were used due to their relatively low sensitivity and specificity [14,15]. 

**Figure 4:**
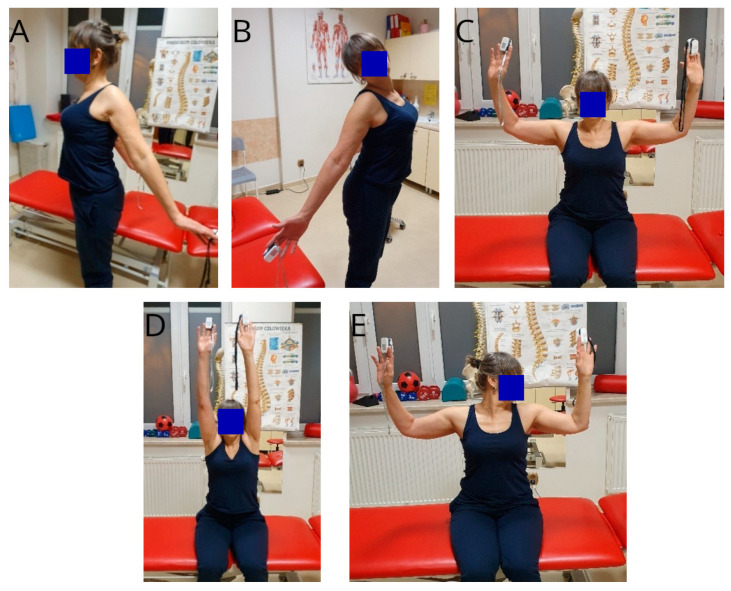
(**A**–**E**), Provocation tests: Eden test (**A**), modified test according to Adson (**B**), Wright test (**C**,**D**), Allen test (**E**), Roos test (**C**). Evaluated parameters: heart rate, pain on NRS scale, feeling of heaviness on NRS scale, discolouration of upper limb.

The following TOS provocation tests were performed as part of a clinical study. Subjects were evaluated in a standing or sitting position (see Figure 4A–E). Bilateral pulse rate measurements were taken with pulse oximeters placed on the index fingers of both hands to more precisely assess the pulse wave [13]. Several provocation tests were used due to their relatively low sensitivity and specificity [14,15].

Eden test: The patient’s scapulae are retracted, and both upper extremities are straight at the elbows and are externally rotated at the scapulo-humeral joint (see Figure 4A). The task is to maintain this position for 1 min [15]. The test specifically evaluates the space between the rib and the clavicula.

Wright test: This test was performed in two positions bilaterally (see Figure 4C,D):

1. The patient’s arms are placed at 90 degrees of abduction and at maximum external rotation, with an elbow flexion of 90 degrees. The position should be maintained for 1 min [15].
2. The patient is asked for maximum abduction with external rotation of the arms and extension of the elbows. The position should be maintained for 1 min.

Allen test: The patient is in a sitting position. The therapist assesses the pulse in the radial artery with a pulse oximeter placed on the index finger. The therapist then places the arms in the position of 90 degrees of abduction, maximum external rotation, and elbow flexion. Then, the patient’s head is rotated to the opposite side. During the test, the patient’s heart rate is continuously monitored. The entire procedure should be repeated for the limbs on both sides of the body (see Figure 4E) [15].

Modified Adson test: The therapist places both upper limbs of the patient in a position of about 30 degrees of abduction, external rotation, and extension at the shoulder joint. The therapist then asks for an extension movement of the head with rotation to the tested limb. The test is performed in the same way on both sides of the body (see Figure 4B). 

Roos test: The patient is in a sitting position. The patient abduces the arms to 90 degrees, sets at maximum external rotation, and bends the elbows to 90 degrees. The patient is then asked to actively clench their hands into fists and relax them. The duration of the test should be 3 min (see Figure 4C) [13,15].

During the performance of all tests, the patient’s heart rate was monitored, recording the occurrence and time of onset of pulse loss, discolouration, and the patient’s symptoms, particularly the feeling of heaviness in the limbs, pain, numbness, using a numerical scale from 0–10, where 0 means no discomfort and 10 means the maximum possible discomfort [16]. In the field of dermatology, there are numerous qualitative assessment tests available that examine skin parameters such as change in colour, extent of rash, or psoriasis [17,18,19]. However, in the case of evaluating bruising of the upper limb in the course of TOS, a suitable measurement tool could not be found. Due to the lack of a generally accepted way of assessing skin colour changes in upper limb venous insufficiency in the literature, our own method of assessment was introduced, in which colour changes in the individual upper limb segments—hand, forearm, and upper arm—were assessed separately. Depending on the extent of discolouration, appropriate scores were given. To differentiate the severity of the discolouration symptom, the following scoring method was adopted: A score of 1—one-third of the upper extremity was bruised (hand, forearm, or arm only). A score of 2—two-thirds of the upper limb was bruised (hand plus forearm, or arm plus forearm, or arm plus hand). A score of 3—bruises were present in the entire upper limb, that is, arm, forearm, and hand. Another variable described during the study was the heart rate score. A score of 10 means that the pulse was present throughout the test. A score of 0 means that the heart rate had already dropped completely before half the test time. A value of 5 was given when the heart rate dropped after half or at the end of the duration of the test.

The tests were carried out twice: during the initial visit and after the physiotherapy intervention. 

### 2.4. Quick DASH Questionnaire

In the next stage of the study, the patient completed the Quick DASH questionnaire. Quick DASH is a shortened version of the DASH questionnaire [12]. It contains 11 items to measure fitness, physical performance, and upper limb discomfort accompanying household chores (e.g., washing walls, mopping floors), carrying a shopping bag or briefcase, complaints during sleep, recreation, etc. Quick DASH also includes two four-point modules that are scored separately. These are the “work” and “sports/playing an instrument” modules. These address the impact of arm, shoulder, or hand problems on the ability to work (including housekeeping if it is the main occupation), and the impact of arm, shoulder, or hand problems on the ability to play an instrument and/or play sports [12,13,14,15,16,17,18,19,20,21,22,23]. Each of the 11 survey items is rated on a point scale from 1 to 5. The lower the total score, the higher the subjective evaluation of the patient. The Quick DASH questionnaire was filled out twice: once at the first meeting and again after 10 physiotherapy sessions.

### 2.5. Intervention

**Figure 5:**
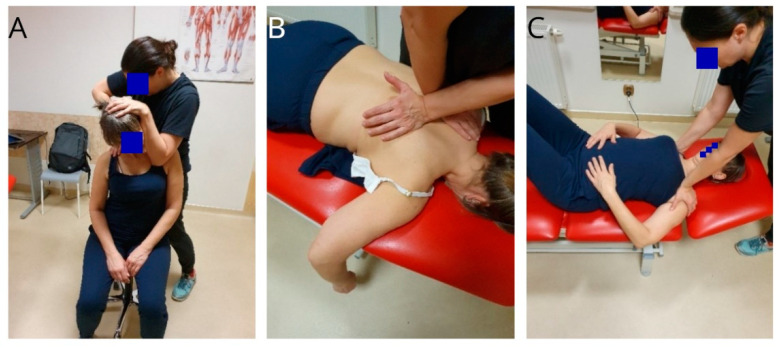
Selected therapeutic techniques: (**A**) mobilisation of the first rib, (**B**) mobilisation of the inter-articular joints in the thoracic region; (**C**) stretching of the minor m. pectoralis.

The physiotherapy intervention consisted of 10 physiotherapy sessions lasting 30 to 45 min, conducted at weekly intervals between November 2023 and January 2024. During the appointment, physical therapy was performed, including manual therapy techniques of mobilisation of the first rib; mobilisation of the upper and middle thoracic region of the intercostal joints and the rib joints; mobilisation of the cervical region; stretching of the pectoralis minor muscle, the anterior scalene muscle, and the middle scalene muscle; dynamic stabilisation exercises for the scapula [11]; corrective Kinesio-taping for alignment of the scapula with respect to the thorax; and soft tissue therapy for the upper quadrant of the body, for example, the subclavius muscle, m. pectoralis minor, m. scalenus anterior, scalenus medius, m. levator scapulae, and m trapezius (Figure 5A–C) [13,24].

Stretching techniques were performed with longitudinal static stretching techniques, in supine positions, moving the initial and final attachments away from each other [25,26]. The stretching pulse was maintained for 10–15 s, with a break for a few seconds and stretching again for a total of about 10–12 min. Then, the stretching exercises were continued at home.

Techniques for the mobilisation of the first rib in the sitting position were performed for about 15–20 min over five physiotherapy sessions. The technique consisted of performing a rib separation at the costo-tranversal joint in the caudal–ventral direction, with a pause of about 10–15 s (Figure 5A) [27]. The thoracic intervertebral joint mobilisation technique (Figure 5B) was performed over five physiotherapy sessions. The technique was performed through transverse processes, with the direction of push being ventral, a holding time of 10–15 s, for a total of about 15–20 min (level of the cervical–thoracic junction and middle section) [20].

### 2.6. Results

**Figure 6:**
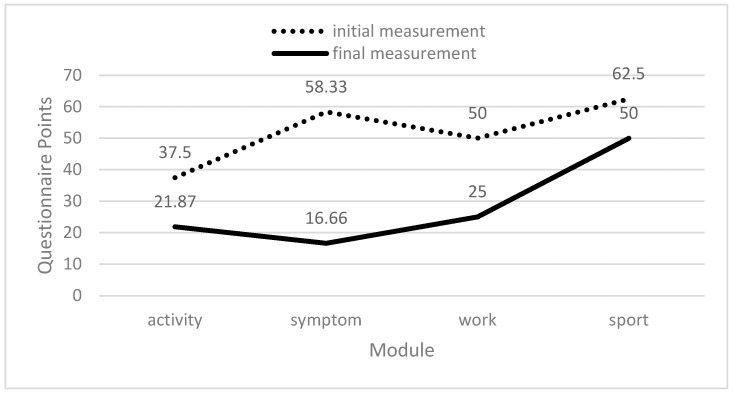
Quick DASH questionnaire results.

According to the Quick DASH questionnaire, the patient achieved a 41.7% improvement in the activity module, a 71.4% improvement in the symptom module, a 50.0% improvement in the work module, and a 20.0% improvement in the sports module, as shown in Figure 6.

**Figure 7:**
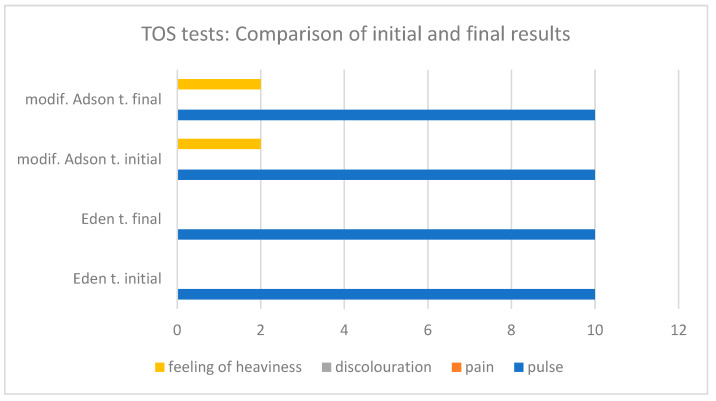
TOS tests: Comparison of initial and final results.

**Figure 8:**
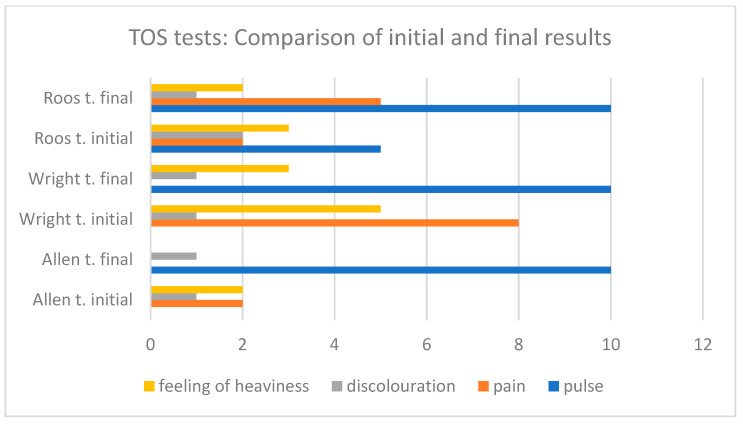
TOS tests: Comparison of initial and final results.

When comparing the period before the intervention with the period after the intervention, improvement was achieved in individual parameters diagnosed with various tests. Pulse palpability increased significantly in the Allen, Wright, and Roos’ tests. In turn, the sensation of pain was eliminated in all provocative tests, except the Roos test, where an increase in pain was reported. The discolouration was only reduced in the Roos test, but the initial level of discomfort was low. The feeling of heaviness was rated as lower in the Allen, Wright, and Roos tests. The results of the tests performed before and after physical therapy are shown in Figure 7 and Figure 8, respectively.

**Figure 9:**
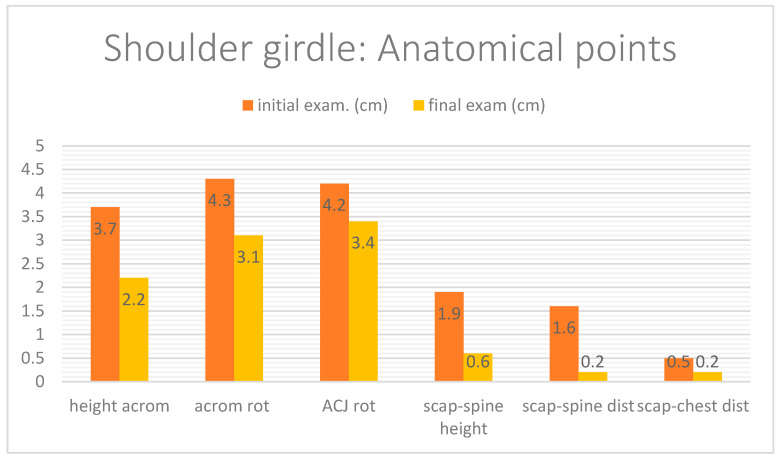
Evaluation of anatomical bone points before and after the intervention. From the left: Height of the acromion process in the frontal plane. Next, rotation at the level of the acromion processes and acromioclavicular joints. The fourth column on the left shows the difference between the right and left sides in the height of the scapula in the frontal plane at the upper angle point. Next, the difference in the distance between the right and left sides of the spine and the lower angle of the scapula. Next, the difference between the sides in the separation of the lower angle from the thorax.

The difference between the height of the acromion process after the intervention was 1.5 cm, which was the highest value of the disproportional reduction achieved. The post-thrombotic side is always placed lower. Then, for rotation at the level of the acromion processes and the acromioclavicular joints, a reduction of 1.2 cm and 0.8 cm, respectively, was obtained. The right side of the body was positioned forward in front of the left side in the transverse plane. When assessing the position of the scapula, the greatest reduction in disproportion was achieved for the difference between the distance between the right and left sides of the spine, which was 1.4 cm. The right scapula was positioned further away from the spine. Taking into account the difference between the scapula’s right and left sides in the height in the frontal plane, a reduction of 1.3 cm was obtained, where the involved side was placed lower. The last parameter was the difference between the sides in the separation of the lower angle from the thorax, which was 0.3 cm. The lower angle on the involved side was more prominent. Baseline and post-intervention measurement data are summarised in Figure 9. It should be noted that abnormalities occur in the position of the scapula in all planes of motion, so the decision was made to implement dynamic stabilisation exercises for the scapula.

## 3. Discussion 

TOS is a complex disorder that involves the compression of the shoulder plexus and/or subclavian vessels. It can usually occur in three locations: in the subclavian space, in the coracoid process, and in the triangular scalene [13]. The clinical manifestation of TOS venous syndrome can present in patients in different ways. The most common symptoms are swelling and a feeling of heaviness in the affected upper extremity, which can be accompanied by moderate pain. In acute VTOS syndrome, symptoms are much more pronounced and include diffuse swelling, bruises, pain, and a palpable clot [28]. Chronic VTOS can cause a network of collateral circulation and venous dilation in the cervical, upper thoracic, and shoulder regions [28]. 

Based on the presence of symptoms, patients with VTOS can be divided into three categories [28].

The first group experiences pressure-related complaints that depend on the position of the upper extremity, but thrombosis occurs. Symptoms occur during specific movements of the upper extremity (known as McCleery syndrome) [28]. In the next category, there is stenosis inside the subclavian vein due to factors such as scalene hypertrophy, increased friction against the first rib, leading to fibrosis and stenosis [28]. In the third group, there is thrombosis that closes the lumen of the vessel, often combined with the appearance of intense physical activity. Another name for this group is Paget–Schroetter syndrome or thrombosis caused by forced exercise (stress thrombosis) [28]. There are cases of venous thrombosis in the upper extremity, in which there is no history of forcible activity or anatomical problems. In this case, the term appears to be idiopathic. In these cases, it appears that a hypercoagulable state associated, for example, with the use of contraceptives or other medications can cause symptoms of VTOS [28].

Referring to articles that describe the cases of patients with VTOS and describe therapeutic interventions, due to the truncated nature of the description, it is not possible to compare the therapeutic intervention performed by our team with other cases in terms of the choice of therapeutic techniques and duration of the intervention [13,24,29]. The proposed management protocol proved to be effective, which encourages continued research in this area. However, the case description is based only on the analysis and correlation between the form of therapy used and the response of the patient’s symptoms, which may be a subjective issue. The lack of a “gold standard” limits the study results to this particular case [29]. The results did not recover full symmetry in the position of the observed anatomical points, nor did the TOS provocation tests. Perhaps the intervention time would require prolongation and continuation of stabilisation training, especially of the serratus anterior muscles, trapezius of the middle and ascending parts, central stabilisation muscles, etc. The time required for the response and improvement of the scapular stabilisers is a minimum of 4–5 months [30]. Scapular stabilisation exercises have two tasks: improving the resting position of the scapula and the work of the scapula during shoulder girdle movements [31,32]. Regarding scapular stabilisation exercises, a common disorder is rotator cuff overload, where scapular stabilisation plays a key role, and the described studies indicate a minimum of one year to evaluate the effectiveness of scapular stabilisation exercises [30]. In future studies on a large population, control tests, including ultrasound evaluation after treatment, will be provided. Before that, the standardisation of TOS ultrasound assessment in those patients is needed, especially the development of relatively measurable and comparable data, which is currently difficult due to the lack of such standards.

To continue the research project, it would be worthwhile to evaluate the long-term effects of therapeutic treatment, including after its termination, and to answer the question of how long it would take to continue treatment if it does not produce the desired changes. It would also be interesting to know whether patients who differ in venous TOS category will respond similarly to therapeutic management.

An important therapeutic option is invasive procedures such as venous stenting, scalenectomy, or total first rib resection [], which increase the chance of asymptomatic further functioning in patients, according to some authors [33,34,35]. However, subclavicular vein stenting is a controversial issue, including the fact that Vuoncino et al. believe that it should not be used even after surgical decompression due to the risk of reocclusion in the stents, especially in young, active people [36]. Surgery is a less controversial option for patients with arterial TOS, especially with repeated vessel trauma due to the nature of their work, for example [36]. Despite the fact that these are minimally invasive/endovascular procedures or thoracic surgeries performed with a small incision, some patients prefer to avoid surgical intervention. For this reason, physiotherapy is the first step in the treatment of patients with TOS, regardless of whether it is an arterial, venous, or exclusively neurological syndrome.

## 4. Conclusions 

This article describes the case of a middle-aged woman diagnosed with a venous form of chronic TOS (VTOS). The above case report proves that it is possible to improve even long-standing complaints. Clinical examination, especially the analysis of asymmetry within bone points, indicates that there may be some postural predisposition to thrombotic disorders in the upper extremity. Based on the analysis of these points, an individual programme of physiotherapy sessions was implemented with good results.

## Data Availability

The data are not publicly available as the information contained could compromise the privacy of patients. Data supporting the findings of this study are available, except for personal information, on request from the corresponding author, Agnieszka Sliwka.
